# Optimization of MechanoATRP through ZnO Loading and Reaction Temperature

**DOI:** 10.1021/acspolymersau.5c00197

**Published:** 2026-04-02

**Authors:** Martin Cvek, Dominik Skopal, Miroslav Mrlik

**Affiliations:** Centre of Polymer Systems, 48362 Tomas Bata University in Zlin, Trida T. Bati 5678, Zlin 760 01, Czech Republic

**Keywords:** ATRP, mechanochemistry, polymers, zinc oxide, reaction kinetics

## Abstract

Mechanically mediated atom transfer radical polymerization (mechanoATRP) has emerged as a promising approach for the controlled synthesis of well-defined polymers, enabled by activator (re)­generation through mechanical stimuli. Besides spatiotemporal control, mechanoATRP enables more uniform generation of electrons owing to the excellent penetration depth of ultrasonic shock waves. However, despite recent progress, the effects of temperature and active particle loading on mechanoredox efficiency remain underexplored. Herein, we present a systematic investigation of how reaction temperature and loading of mechanotransducers jointly govern polymerization kinetics and control in the mechanoATRP of methyl acrylate. ZnO nanocrystals, synthesized via a rapid microwave (MW)-assisted route, were employed as active agents. To decouple thermal and mechanochemical contributions, we designed an ultrasonic setup capable of maintaining precise isothermal conditions during mechanoATRP. By combining experimental techniques, kinetic analysis, and Arrhenius evaluation, the operational limits of mechanoATRP were identified, and the apparent propagation rate constant and apparent activation energy were determined and correlated with both temperature and ZnO loading. The results indicated that the apparent activation energy is strongly dependent on ZnO loading, reflecting changes in the efficiency of mechanochemical catalyst activation. The ZnO loading of 1.0 wt % was identified as the optimum dosing across the temperature range of 25–45 °C, providing high polymerization rates and excellent control over the kinetics. This study provides quantitative insight into the mechanoredox activation mechanism, yielding optimizations of mechanoATRP and advancing its potential toward scalability and energy efficiency.

## Graphical abstract

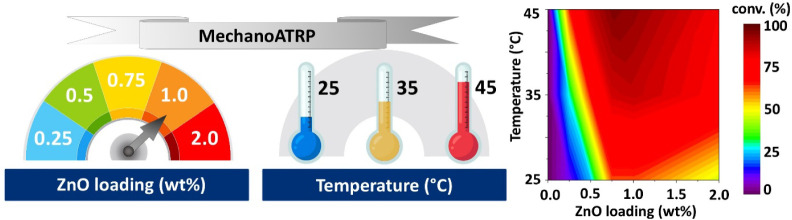

## 1 Introduction

Reversible-deactivation radical polymerizations (RDRPs) are a powerful class of synthetic techniques that enable access to well-defined polymers with predetermined molecular weights, high chain-end fidelity, tailored composition and architectures, and narrow molecular-weight distributions.^1^ Among these, atom transfer radical polymerization (ATRP) represents one of the most versatile and robust RDRP approaches, operating through a dynamic equilibrium between propagating alkyl radicals and dormant alkyl halide species. Mechanistically, ATRP is mediated by redox-active transition metal complexes, where a lower-oxidation-state complex, typically Cu­(I)/L, acts as the activator (generating radicals via halogen abstraction), while the corresponding higher oxidation state halide-bearing complex, typically X–Cu­(II)/L, serves as the deactivator, reforming the dormant species.^2^ Recent advances, including the development of highly active catalysts operating at a billion times lower concentrations^3^ and the implementation of nontoxic solvents, have significantly reduced the environmental impact of ATRP.^4^ Nonetheless, even with highly active catalysts, radical termination events are unavoidable, causing the irreversible conversion of Cu­(I)/L activators to deactivators, and thus, premature cessation of polymerization and incomplete monomer conversion.^5^ For this reason, various strategies enabling continuous regeneration of Cu­(I)/L activators have been developed to sustain the ATRP equilibrium. In regenerative ATRP systems, the latent Cu­(II)/L complexes can be reduced either chemically or through externally applied physical stimuli. Chemical regeneration approaches involve the use of reducing agents such as ascorbic acid in activators regenerated by electron transfer (ARGET) ATRP,^6^ conventional radical initiators such as azobis­(isobutyronitrile) (AIBN) in initiators for continuous activator regeneration (ICAR) ATRP,^7^ or zerovalent metals such as metallic copper (Cu^0^) in supplemental activator and reducing agent (SARA) ATRP.^8^ More recently, externally applied physical stimuli have been utilized to promote activator (re)­generation,^9^ including light irradiation in photochemically mediated ATRP (photoATRP),^10^ electric current in electrochemically mediated ATRP (eATRP),^11^ and mechanical force in mechanically mediated ATRP (mechanoATRP).^12^ In addition to eliminating the need for reducing chemicals, externally controlled ATRP affords on-demand spatiotemporal control of the polymerization process.^13^

Among externally driven chemical processes, mechanochemical activation has emerged as an intriguing strategy for driving polymer transformations.^9,14^ The use of mechanical forces in polymer sciences has historically been linked to polymer degradation and mechanical failure, primarily through processes such as chain scission under stress. However, this perception has evolved, and mechanical force is increasingly viewed as a useful tool for inducing specific and constructive changes in polymer structures, mainly due to the concept of force-sensitive molecular units called “mechanophores”.^15,16^ In the realm of RDRPs, mechanical forces were first introduced in 2016 to reduce X–Cu­(II)/L complexes, thereby activating the ATRP process.^12^ The system required the addition of commercial tetragonal large barium titanate (BaTiO_3_) nanoparticles (200 nm), which provided piezocatalytic reduction of Cu­(II) upon ultrasonication that successfully mediated the polymerization. Further advancements, based on optimizing the piezocatalytic system, allowed the Cu-catalyst loading to be decreased to ppm levels while attaining even higher molecular weights of the resulting polymer.^17^ Later on, the application of zinc oxide (ZnO) nanoparticles enabled a substantial reduction in the required loading of active agents (to below 1.0 wt % relative to the reaction mixture). This improvement was attributed to the higher specific surface area of ZnO and its stronger interactions with X–Cu­(II)/L complexes, which enhanced the efficiency of mechanoredox activation.^13,18^ In addition, the choice of solvent can significantly influence mechanoATRP performance. Polar solvents such as dimethyl sulfoxide (DMSO) have been reported to promote efficient polymerization by solubilizing the Cu catalyst complex and facilitating electron transfer process, enabling high (>90%) monomer conversions.^19^ Recent research has increasingly focused on designing suitable piezocatalysts for mechanoATRP. Notably, the dimensional architecture of ZnO materials was found to strongly influence their catalytic performance; specifically, one-dimensional (1D) ZnO structures with higher aspect ratios exhibited superior piezocatalytic activity compared to their zero-dimensional (0D) analogs prepared via chemical bath deposition.^20^ Furthermore, ZnO/BaTiO_3_ heterostructures demonstrated enhanced polymerization rates, attributed to more efficient piezoelectrically induced charge separation and transfer.^21^ Beyond conventional piezoelectrics, alternative mechanotransducers have also been explored for mechanoATRP, including the CaZnOS–ZnS–SrZnOS: Mn^2+^ ternary heterojunction, which facilitated mechanoactivation during ATRP to produce mechanoluminescent composites,^22^ and Bi_0.5_Na_0.5_TiO_3_, identified as a promising alternative to traditional materials.^23^

Ultrasound represents a particularly attractive external stimulus capable of generating both chemical and mechanical effects that are widely used in polymer chemistry.^24^ In the low-frequency range (20–40 kHz), ultrasound can activate piezoelectric materials by inducing mechanical strain, which generates strain-induced voltages capable of reducing metal catalysts and thereby driving redox-mediated ATRP processes.^25^ However, ultrasonic agitation also induces cavitational phenomena and heat generation, which increase both local and bulk temperatures of the reaction medium and make precise temperature control inherently challenging.^25,26^ Consequently, in most mechanoATRP studies the reaction temperature either increased during sonication (typically without precise control, e.g., 20–30 °C), or was maintained at a relatively high constant temperature (e.g., 50 °C) to compensate for this effect.^19^ Therefore, systematic investigations quantifying the influence of temperature on the kinetics of mechanoATRP remain scarce.

To address this limitation, we coupled an ultrasonic bath with a thermostatic control system, enabling polymerizations to be performed under strictly defined isothermal conditions. This setup allowed us to elucidate correlations between reaction kinetics, ZnO loading, and temperature, and to determine the apparent activation energy of the mechanochemically mediated ATRP, thereby providing quantitative insights for process optimization. As the piezotransducer, we employed ZnO nanocrystals synthesized via microwave (MW)-assisted methods, offering rapid, energy-efficient, and morphology-tunable preparation of high-quality ZnO.^27^ These ZnO nanostructures have previously demonstrated remarkable effectiveness in mechanoATRP,^28^ as well as in optoelectronic^29^ and antibacterial applications,^30^ providing an excellent platform for mechanistic and kinetic studies. Overall, this work systematically investigates the effects of temperature and ZnO loading on polymerization kinetics, providing new insights into the operational limits of mechanoATRP and expanding its scope for practical applicability. Importantly, the study demonstrates that the apparent activation energy of mechanoATRP is strongly influenced by the loading of ZnO nanocrystals, suggesting that the efficiency of mechanochemical catalyst activation plays a key role in governing the polymerization kinetics.

## 2 Methodology

### 2.1 Materials

All chemicals were obtained from commercial suppliers and employed without further purification unless stated otherwise. Zinc acetate dihydrate (ZAD, p.a.), diethylene glycol (DEG, ≥99.0%), oleic acid (OA, ≥99.0%), and methanol (≥99.6%) were purchased from Sigma-Aldrich (USA) and used for the synthesis of ZnO nanocrystals. Reagents utilized in the mechanoATRP process included methyl acrylate (MA, 99%), ethyl-α-bromoisobutyrate (EBiB, 98%), tris­(2-pyridylmethyl)­amine (TPMA, 98%), and copper­(II) bromide (CuBr_2_, 99%), all sourced from Sigma-Aldrich (USA). Before use, monomers were purified by passing through a column of activated aluminum oxide (Brockmann I) to remove inhibitors. Solvents such as dimethyl sulfoxide (DMSO, ≥99.7%), tetrahydrofuran (THF, anhydrous ≥99.9%), and deuterated chloroform (CDCl_3_, 99.8%) were also obtained from Sigma-Aldrich (USA).

### 2.2 Synthesis of ZnO Nanocrystals

The ZnO nanocrystals were prepared via MW-assisted synthesis, as reported in our preceding papers.^28,30^ In brief, 32 mmol (7.02 g) of ZAD precursor was dispersed in 50 mL of DEG, serving as a medium. Subsequently, OA (2.3 mmol, 0.660 g) was introduced as a capping agent. The resulting mixture was sonicated in a Teflon-lined reactor for 10 min to ensure uniform dispersion and homogeneity, and subjected to MW irradiation using a Magnum II reactor (Ertec, Poland). The irradiation power and reaction time were set to 100% intensity and 15 min, respectively. During the process, the reaction temperature rapidly increased and was maintained within the range of 245–250 °C, promoting nucleation and controlled growth of the ZnO nanocrystals. Upon completion, the reactor was cooled to 45 °C via an in-built circulating water system. The resulting ZnO product was washed thoroughly with methanol, separated by centrifugation (5 min, 6000 rpm) using EBA21 centrifuge (Hettich, Germany), and dried overnight under reduced pressure (150 mbar).

### 2.3 General Procedure for MechanoATRP with ZnO

A typical mechanoATRP procedure was employed to ensure consistency with established literature protocols and facilitate reproducibility across research groups.^18,28,31^ In brief, the reaction mixture consisted of MA (1.92 mL, 21.2 mmol, 100 equiv), EBiB (31.1 μL, 0.21 mmol, 1 equiv), TPMA (9.8 mg, 33.9 μmol, 0.16 equiv), CuBr_2_ (1.9 mg, 8.5 μmol, 0.04 equiv), and DMSO (50 vol %). Due to the low quantities required, TPMA and CuBr_2_ were dosed from stock solutions to enhance dosing accuracy. ZnO nanocrystals were incorporated into the ATRP mixture in concentrations of 0.25, 0.50, 0.75, 1.00, and 2.00 wt % relative to the combined mass of monomer and solvent (DMSO). After dosing all components, the dispersion was homogenized using a vortex mixer (TX4, Velp Scientifica, Italy), followed by deoxygenation under an argon atmosphere for 5 min. Polymerization was initiated by subjecting the reaction vial to ultrasonic shock waves in an ultrasonic bath (T490 DH, Elma Transsonic Digital, Germany) operating at a frequency of 40 kHz. The reaction temperature was precisely controlled at 25 °C, 35 °C, or 45 °C using a temperature regulator (CC-K6, Huber Pilot One, Germany) coupled to the ultrasonic bath in a custom-built setup (Figure 1). This temperature range was selected to ensure experimental feasibility, considering the freezing point of DMSO (19 °C), and to minimize competing thermal radical generation (above 45 °C) that could interfere with the mechanochemical catalyst regeneration process. To ensure experimental reproducibility, the reaction vial was placed in a polystyrene (PS)-based holder, maintaining a fixed position within the bath. Each mechanoATRP experiment was conducted over a period of 6 h. Reaction aliquots (100 μL) were collected at 2-h intervals (at 2, 4, and 6 h) for subsequent analysis. Reference experiments were performed under identical conditions and temperatures but without the addition of ZnO nanocrystals. All reactions were performed in 4.8 mL closed-cap vials with a 20% (v/v) headspace, resulting in an effective reaction volume of 3.84 mL.

**Figure 1:**
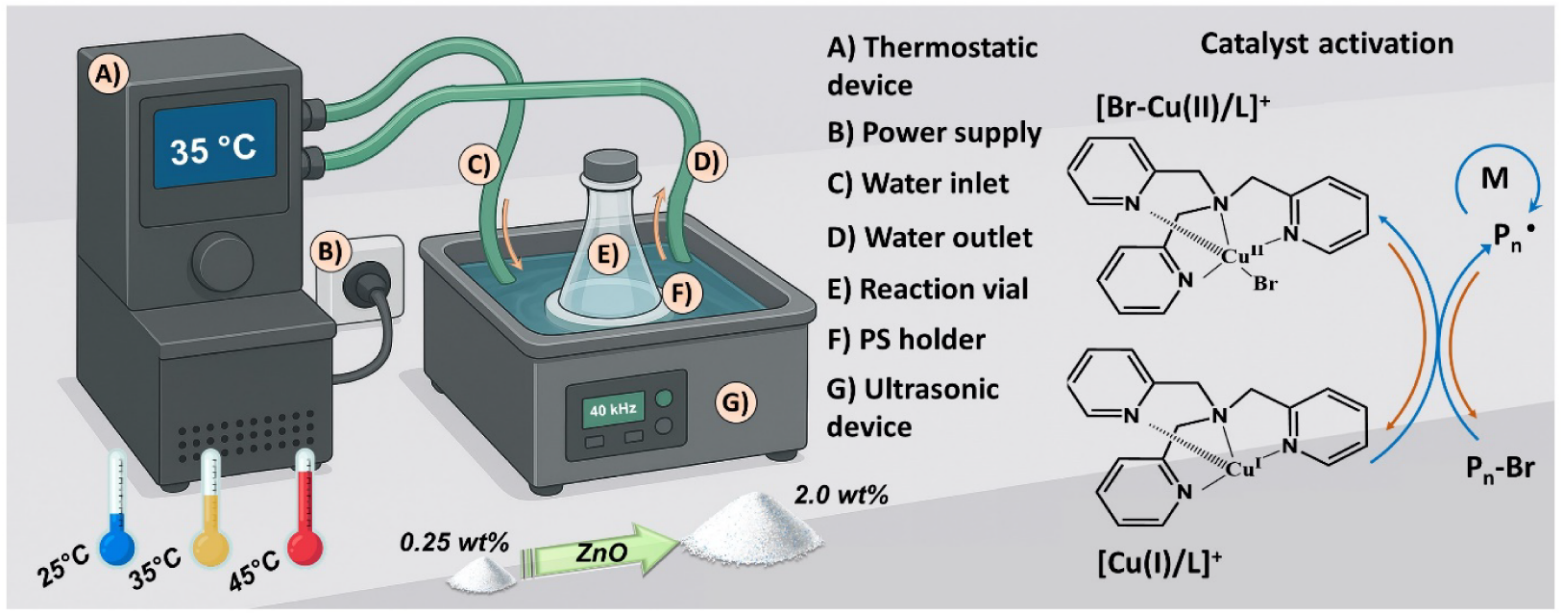
Scheme illustrating the experimental setup employed for mechanoATRP experiments at isothermal conditions, together with a fundamental mechanism of catalyst activation.

### 2.4 NMR Characterization

Reaction aliquots were dissolved in CDCl_3_ and passed through a column of alumina and a polytetrafluoroethylene (PTFE) syringe filter (pore size 0.22 μm) to prepare samples for ^1^H NMR analysis. The ^1^H NMR spectra were recorded at laboratory temperature on an ECZ400R/S3 spectrometer (JEOL, Japan) equipped with a JASTEC 400/54 mm magnet and a ROYAL Probe HFX (5 mm), operating at 400 MHz. Monomer conversion was calculated from the obtained spectra. The representative procedure for calculating monomer conversion from ^1^H NMR spectra is provided in Figure S1 (Supporting Information).

### 2.5 GPC Characterization

For GPC analysis, each reaction aliquot was dissolved in tetrahydrofuran (THF) stabilized with butylated hydroxytoluene (BHT, 240 mg/L) and filtered through a column of alumina and a PTFE syringe filter (pore size 0.22 μm). GPC was performed on a Waters HPLC system equipped with a model e2695 separations module and a model 2414 differential refractometer (Waters Corporation, USA). The separation was carried out using a series of mixed-bed columns (Polymer Laboratories, UK): PLgel-Mixed-A (300 × 7.5 mm, 20 μm), PLgel-Mixed-B (300 × 7.5 mm, 10 μm), and PLgel-Mixed-D (300 × 7.5 mm, 5 μm). THF stabilized with BHT was used as the mobile phase at a flow rate of 1.0 mL/min, with an injection volume of 100 μL at 40 °C. The weight-average molar mass (Mw), number-average molar mass (*M*_n_), and dispersity index (*Đ* = *M*_w_/*M*_n_) were calculated from the resulting GPC traces.

## 3 Results and Discussion

### 3.1 General Mechanism of MechanoATRP with ZnO Nanocrystals

The mechanoATRP process relies on the presence of ZnO nanocrystals, which enable mechanochemical (re)­generation of the ATRP activator species under ultrasonic stimulation. As illustrated in Figure 2, mechanical deformation of ZnO nanocrystals generates localized charges that promote electron transfer to the Cu­(II)/L complexes, producing the catalytically active Cu­(I)/L species. The Cu­(I)/L complex subsequently activates the dormant alkyl bromide initiator or polymer chain end (P_n_–X), generating a propagating macroradical (P_n_•) while regenerating the Cu­(II)/L deactivator. The resulting dynamic activation–deactivation equilibrium maintains a low concentration of propagating radicals, enabling controlled polymer growth characteristic of ATRP.^18,20,21^

**Figure 2:**
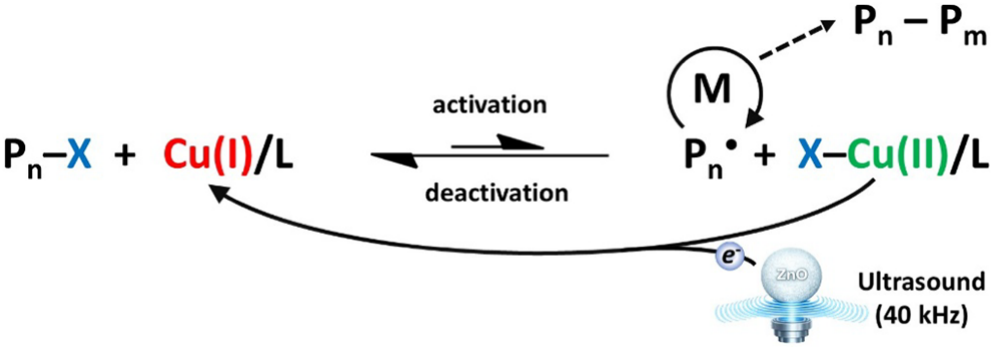
General mechanism of ZnO-mediated mechanoATRP, involving mechanochemically induced reduction of Cu­(II)/L to Cu­(I)/L and the establishment of the ATRP activation–deactivation equilibrium.

### 3.2 Effects of ZnO Loading on MechanoATRP at 35 °C

ZnO nanoparticles are well-established as effective piezotransducers for mediating mechanoATRP. However, the optimization of their loading has not been systematically investigated. In this study, we adopted the reaction formulation [MA]_0_/[EBiB]_0_/[CuBr_2_]_0_/[TPMA]_0_ = 100/1/0.04/0.16, which recently demonstrated promising performance in terms of both reaction kinetics (conv. 54% after 5 h) and control over the polymerization (Mn of 5250, *Đ* of 1.10) when catalyzed with 0.5 wt % of ZnO.^28^ Such mechanoATRP requires an excess of ligand, typically, a CuBr_2_/ligand ratio of 1:4, due to the reliance on amine-mediated regeneration of the active Cu­(I) catalyst species. To evaluate and optimize the catalytic efficiency of custom-made ZnO nanocrystals, their loading was varied between 0.25 and 2.0 wt %. The corresponding polymerization outcomes, as determined by ^1^H NMR spectroscopy and GPC, are summarized in Table 1 and Figure 3.

**Table 1:** MechanoATRP of MA Catalyzed by Various Amounts of ZnO Nanocrystals at 35 °C

Entry^a^	**ZnO (wt %)**	**Time (h)**	Conv.^b^ **(%)**	***M***_**n,th**_ ^c^ (g/mol)	***M***_**n,GPC**_ ^d^(g/mol)	***Đ*** ^d^ (−)
1	0.00	6	–	–	–	–
2	0.25	2	–	–	–	–
		4	8	900	700	1.41
		6	28	2600	2636	1.16
3	0.50	2	22	2100	2100	1.18
		4	54	4900	5100	1.10
		6	67	6000	6300	1.09
4	0.75	2	37	3400	3500	1.23
		4	79	7000	5800	1.14
		6	85	7500	7000	1.13
5	1.00	2	43	3900	3900	1.15
		4	72	6400	6900	1.09
		6	87	7700	7800	1.09
6	2.00	2	44	4000	4100	1.25
		4	68	6100	6100	1.19
		6	78	6900	7100	1.16

a Reaction conditions: [MA]_0_/[EBiB]_0_/[CuBr_2_]_0_/[TPMA]_0_ = 100/1/0.04/0.16 in 50% (v/v) DMSO, argon-purged (5 min); closed-capped reactor with 20% (v/v) headspace; ultrasound source (40 kHz) at 35 °C.

b Determined from ^1^H NMR spectra (CDCl_3_ as solvent).

c Calculated following the equation (*M*_n,th_ = *M*_EBiB_ + [MA]_0_/[EBiB]_0_ × conversion × *M*_MA_).

d Determined by GPC analysis (THF as eluent), calibrated to a linear PMMA standard.

**Figure 3:**
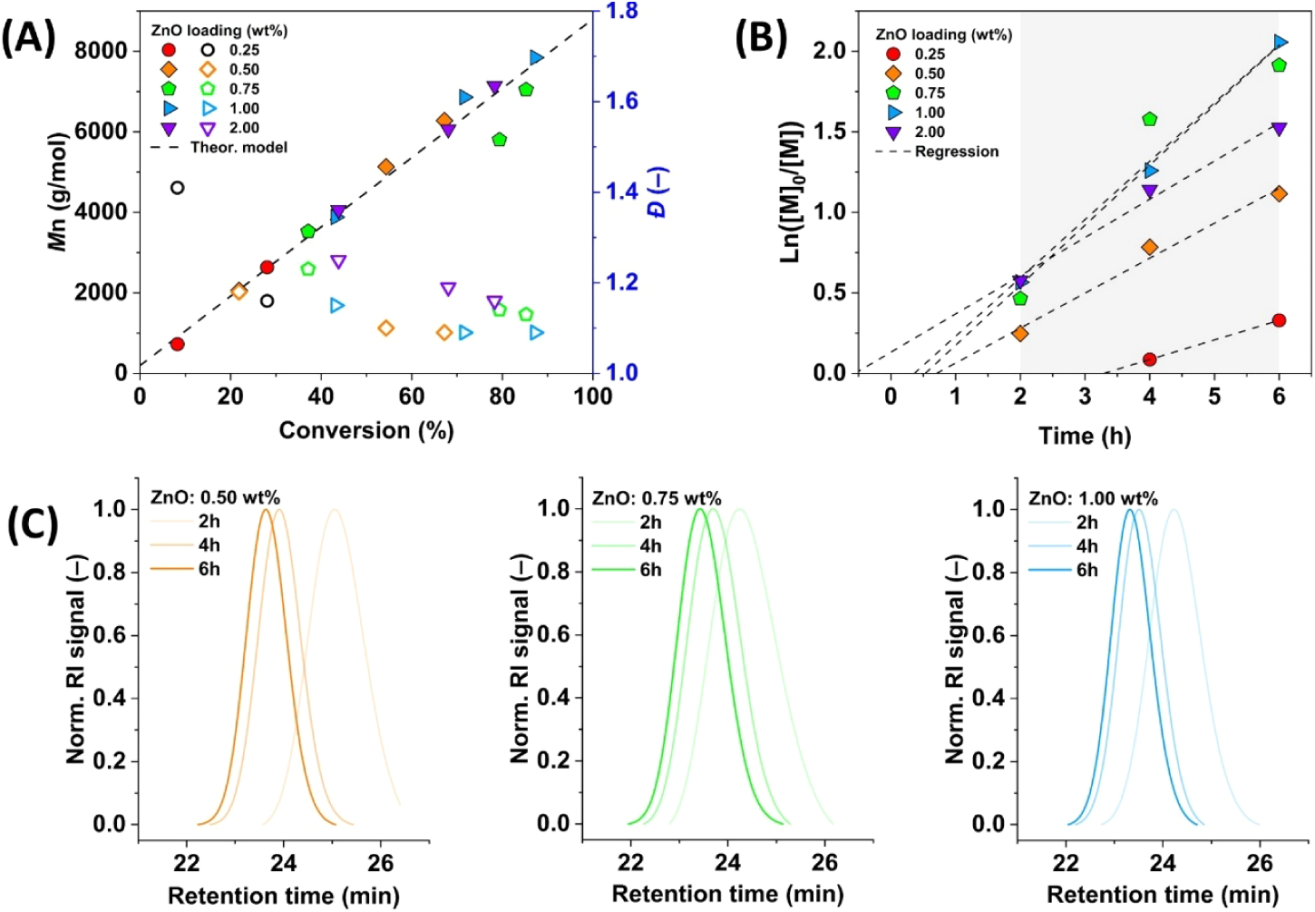
(A) The evolution of Mn (solid symbols) and *Đ* (open symbols) with monomer conversion, (B) semilogarithmic kinetic plots with the fitted region (2–6 h) used for *k*_app_ determination highlighted, and (C) the representative GPC traces for mechanoATRP of MA catalyzed with ZnO nanocrystal loadings of 0.50, 0.75, and 1.0 wt %. The corresponding GPC traces obtained for 0.25 and 2.0 wt % ZnO loadings are provided in Figure S2 (Supporting Information). Reaction conditions: [MA]_0_/[EBiB]_0_/[CuBr_2_]_0_/[TPMA]_0_ = 100/1/0.04/0.16 in 50% (v/v) DMSO, argon-purged (5 min); closed-capped reactor with 20% (v/v) headspace; ultrasound source (40 kHz) at 35 °C.

In the absence of ZnO nanocrystals (Table 1, Entry 1), no monomer conversion was observed, confirming that thermal initiation did not occur under the applied conditions. Upon introduction of 0.25 wt % ZnO (Table 1, Entry 2), the reaction exhibited a prolonged induction period of approximately 3 h, during which no detectable polymerization took place. However, after 4 h, a measurable conversion was observed, which further increased to 28% at 6 h. As the ZnO loading was increased (Table 1, Entries 3–6), a higher monomer conversion was achieved for the corresponding withdrawal times, consistent with the formation of polymers of higher molecular weight. This trend was corroborated by GPC analysis (Figure 2C), where the elution profiles progressively shifted toward shorter retention times, confirming an increase in the molecular weight of the synthesized poly­(methyl acrylate) (PMA). In all cases, the GPC traces of resultant PMA were monomodal and narrow, reflecting the high degree of control characteristic of a well-regulated mechanoATRP process. Upon closer examination, however, a nonlinear relationship between ZnO loading and polymerization efficiency, in terms of conversion, was revealed. For clarity, the relationship between ZnO loading and monomer conversion at fixed reaction times (2, 4, and 6 h) is presented in Figure S3B (Supporting Information). Notably, significant improvements in conversion were achieved at ZnO loadings of 0.5 and 0.75 wt % (Table 1, Entries 3 and 4), likely due to more efficient regeneration of the active Cu­(I)/ligand species through enhanced mechanochemical activation. The most efficient polymerization was observed at a ZnO loading of 1 wt % (Table 1, Entry 5), achieving 43% conversion within 2 h and reaching 87% conversion after 6 h, with an exceptionally low dispersity (*Đ* = 1.09). Interestingly, further increasing the ZnO loading to 2.0 wt % (Table 1, Entry 6) resulted in comparable conversion at short reaction times but a noticeable decline in polymerization efficiency after prolonged reaction (6 h), accompanied by a slight broadening of the molecular weight distribution. This reduction in performance was attributed to the aggregation of ZnO at higher concentrations, leading to a nonhomogeneous dispersion and a decreased efficiency of electron transfer.

### 3.3 Effects of Temperature on MechanoATRP

To investigate the influence of temperature on the efficiency of mechanoATRP, the identical polymerization formulation [MA]_0_/[EBiB]_0_/[CuBr_2_]_0_/[TPMA]_0_ = 100/1/0.04/0.16 was tested at 25 °C and 45 °C, and the results are summarized in Tables 2 and 3, respectively. As shown, similar trends in polymerization behavior were observed; however, the polymerization kinetics and control were notably affected by the temperature. Expectedly, the reference reaction performed at 25 °C (Table 2, Entry 7) yielded no monomer conversion, confirming the absence of thermally initiated polymerization. When using 0.25 wt % of ZnO nanocrystals (Table 2, Entry 8), the polymerization proceeded very slowly, yielding only 12% conversion after 6 h, which indicated insufficient mechanochemical activation. Upon increasing the ZnO loading to 0.5 wt % (Table 2, Entry 9), the reaction showed modest improvements, reaching a conversion of 24% after 6 h. Although the molecular weights remained low, the dispersity was within a moderate range (*Đ* = 1.22–1.36), indicating the onset of controlled polymerization. A further increase of ZnO nanocrystals to 0.75–1.0 wt % ZnO (Table 2, Entries 10 and 11) significantly enhanced the reaction rate and control, both yielding 63% conversion after 6 h and a desirably narrow dispersity (*Đ* = 1.13–1.14). This trend is also evident from Figure S3A (Supporting Information) showcasing the relationship between monomer conversion and ZnO loading. However, the reaction with 1.0 wt % ZnO loading exhibited a slower onset, suggesting a less efficient activation–deactivation equilibrium at shorter reaction times, which might be attributed to the greater ZnO aggregation at lower temperature, hence a lower reproducibility. When using 2.0 wt % of ZnO (Table 2, Entry 12), the conversion reached only 46% after 6 h, with broader molecular weight distributions (*Đ* = 1.33–1.40). Such behavior can be associated with excessive generation of Cu­(I)/L activator species, which increased the probability of termination events,^32,33^ similar to the behavior observed in overstimulated photoATRP.^34^ In addition, ZnO aggregation at elevated loadings may introduce spatial heterogeneity into the catalytic environment. In mechanoATRP systems, aggregated nanoparticles subjected to ultrasonication can generate localized electric fields, which may promote nonuniform reduction of Cu­(II)/L complexes in the vicinity of particle surfaces.^18^ As a consequence, aggregation may create spatially confined regions with locally elevated activator concentration, increasing the probability of termination events. Therefore, the observed loss of control at 2.0 wt % ZnO most plausibly arose from a combined effect of locally intensified mechanoredox activation and spatially heterogeneous catalyst reduction. Moreover, as seen in Figure 4A, the reactions yielded PMA with the higher *M*_n,GPC_ compared to the theoretical prediction, which, analogously to thermal ATRP,^35^ could be explained by decreased initiation efficiency at low temperature, resulting in fewer but longer polymer chains.

**Table 2:** MechanoATRP of MA Catalyzed by Various Amounts of ZnO Nanocrystals at 25 °C

Entry^a^	**ZnO (wt %)**	**Time (h)**	Conv.^b^ **(%)**	***M***_**n,th**_ ^c^ (g/mol)	***M***_**n,GPC**_ ^d^ (g/mol)	***Đ*** ^d^ (−)
7	0.00	6	–	–	–	–
8	0.25	2	–	–	–	–
		4	8	900	–	–
		6	12	1200	2100	1.31
9	0.50	2	9	1000	1200	1.36
		4	18	1700	2200	1.22
		6	24	2200	2600	1.28
10	0.75	2	34	3100	3700	1.17
		4	51	4600	5200	1.14
		6	63	5600	6100	1.13
11	1.00	2	18	1700	2100	1.20
		4	51	4600	5100	1.17
		6	63	5600	6300	1.14
12	2.00	2	22	2100	3300	1.40
		4	32	2900	3800	1.36
		6	46	4200	4900	1.33

a Reaction conditions: [MA]_0_/[EBiB]_0_/[CuBr_2_]_0_/[TPMA]_0_ = 100/1/0.04/0.16 in 50% (v/v) DMSO, argon-purged (5 min); closed-capped reactor with 20% (v/v) headspace; ultrasound source (40 kHz) at 25 °C.

b Determined from ^1^H NMR spectra (CDCl_3_ as solvent).

c Calculated following the equation (*M*_n,th_ = *M*_EBiB_ + [MA]_0_/[EBiB]_0_ × conversion × *M*_MA_).

d Determined by GPC analysis (THF as eluent), calibrated to a linear PMMA standard.

**Table 3:** MechanoATRP of MA Catalyzed by Various Amounts of ZnO Nanocrystals at 45 °C

Entry^a^	**ZnO (wt %)**	**Time (h)**	Conv.^b^ **(%)**	***M***_**n,th**_ ^c^ (g/mol)	***M***_**n,GPC**_ ^d^ (g/mol)	***Đ*** ^d^ (−)
13	0.00	6	–	–	–	–
14	0.25	2	6	700	<800	N.D.
		4	19	1900	2100	2.17
		6	65	5800	6000	1.41
15	0.50	2	24	2300	3100	1.41
		4	75	6700	6800	1.22
		6	87	7700	7900	1.18
16	0.75	2	77	6800	6900	1.10
		4	81	8100	8200	1.09
		6	95	8300	8500	1.09
17	1.00	2	68	6000	6300	1.13
		4	89	7800	8300	1.11
		6	93	8200	8500	1.11
18	2.00	2	38	3500	5000	1.40
		4	74	6600	8200	1.24
		6	80	7100	8700	1.23

a Reaction conditions: [MA]_0_/[EBiB]_0_/[CuBr_2_]_0_/[TPMA]_0_ = 100/1/0.04/0.16 in 50% (v/v) DMSO, argon-purged (5 min); closed-capped reactor with 20% (v/v) headspace; ultrasound source (40 kHz) at 45 °C.

b Determined from ^1^H NMR spectra (CDCl_3_ as solvent).

c Calculated following the equation (*M*_n,th_ = *M*_EBiB_ + [MA]_0_/[EBiB]_0_ × conversion × *M*_MA_).

d Determined by GPC analysis (THF as eluent), calibrated to a linear PMMA standard.

**Figure 4:**
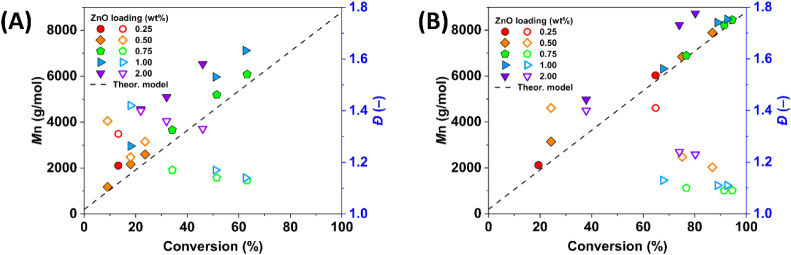
Evolution of Mn (solid symbols) and *Đ* (open symbols) with conversion for mechanoATRP of MA catalyzed with various loadings of ZnO nanocrystals at (A) 25 °C and (B) 45 °C. The corresponding semilogarithmic kinetic plots with the fitted region (2–6 h) used for *k*_app_ determination highlighted are shown in Figure S4 (Supporting Information). Reaction conditions: [MA]_0_/[EBiB]_0_/[CuBr_2_]_0_/[TPMA]_0_ = 100/1/0.04/0.16 in 50% (v/v) DMSO, argon-purged (5 min); closed-capped reactor with 20% (v/v) headspace; ultrasound source (40 kHz).

After increasing the temperature to 45 °C (Table 3), polymerization of MA via mechanoATRP proceeded much more effectively. Despite a relatively high temperature, no monomer conversion was detected in the absence of ZnO nanocrystals (Table 3, Entry 13). Loading of 0.25 wt % ZnO (Table 3, Entry 14) yielded a dramatic improvement compared to the analogous situation at 25 °C, achieving 65% conversion after 6 h. However, an unsatisfactory *Đ* value indicated a poor control over the reaction, likely due to insufficient regulation of radical concentration. From a mechanistic perspective, controlled ATRP relies on a rapid activation–deactivation equilibrium that maintains a very low concentration of propagating radicals, thereby minimizing termination reactions and ensuring narrow molecular weight distribution.^32^ In the present system, at low ZnO loading the mechanochemical reduction of Cu­(II)/L to Cu­(I)/L likely proceeded relatively slowly due to the limited number of piezoelectrically active interfaces. At elevated temperature, however, the propagation rate constant increased significantly, allowing substantial monomer conversion even at relatively low activator concentration. At the same time, the slower establishment of the activation–deactivation equilibrium increased the probability of termination events, resulting in moderate broadening of dispersity and a potential reduction in chain-end fidelity (cf. Table 1, Entry 2 vs Table 3, Entry 14).^2,35^ Optimal performance was observed with 0.75–1.0 wt % ZnO loadings (Table 3, Entries 16 and 17, and Figure S3C), where conversions exceeded 90% after 6 h, with narrow and consistent dispersities (*Đ* = 1.09–1.11). The ZnO loading, within the range of 0.25–1.0 wt %, provided PMA with molecular weights closely matching the theoretical values, underscoring excellent control over the mechanoATRP process. In contrast, when using a ZnO loading of 2.0 wt % (Table 3, Entry 18), conversion remained relatively high; however, the dispersity slightly increased (*Đ* = 1.23). As discussed above, excessive mechanochemical activation at high ZnO loadings may increase the probability of termination events due to locally elevated Cu­(I)/L concentrations. At the same time, spatially heterogeneous catalyst activation may lead to uneven initiation efficiency, resulting in a broader distribution of PMA chain lengths and apparent deviation of Mn from theoretical prediction (Figure 4B). Overall, elevated temperatures (35 and 45 °C) accelerated the mechanoATRP kinetics while enabling slightly better control with low dispersities compared to polymerizations conducted at 25 °C (cf. Tables 1–3), suggesting that chain-end fidelity was likely preserved to a significant extent under these conditions.

### 3.4 Assessment of Testing Parameters

The data clearly demonstrate that the efficiency of the mechanoATRP process is strongly influenced by both the reaction temperature and the ZnO loading. The correlation between these parameters is shown in Figure 5. As observed, an increase in temperature accelerated the polymerization kinetics, but in combination with a high ZnO loading (2.0 wt %), compromised the ATRP equilibrium, leading to polymers with broader molecular weight distributions (*Đ* > 1.25). Conversely, reactions conducted at low temperatures and low ZnO loading did not generate sufficient mechanochemical activation to mediate the ATRP process. To gain deeper insight into the kinetic behavior, the apparent propagation rate constants (*k*_app_) were determined using the following equation:

1 $$\ln\left(\frac{{\left[M\right]}_{0}}{\left[M\right]}\right)=k_{\mathrm{app}}t$$

where [*M*]_0_ and [*M*] are initial and time-dependent monomer concentrations, respectively, and *t* is the reaction time.^36,37^ In well-controlled ATRP systems, the semilogarithmic kinetic plot of ln­([*M*]_0_/[*M*]) versus time typically exhibits linear behavior, reflecting the first-order kinetics with respect to monomer concentration and an approximately constant concentration of the growing radicals maintained by the dynamic activation–deactivation equilibrium between Cu­(II)/Cu­(I) complexes.^17,38^ However, before this equilibrium is fully established, the rate of polymerization may deviate from linearity because a suitable concentration of the Cu­(I)/L activator species is not yet generated by external stimulus, in this case mechanical activation. This phenomenon is often accompanied by the onset of an induction period.^17,28^ For this reason, to avoid bias from the initial transient state that involves catalyst adsorption on ZnO surfaces, diffusion limitations, and incomplete activation, *k*_app_ was extracted from the linear region of the kinetic plots (*t* ranging from 2 to 6 h), representing the steady-state of the polymerization. The activation parameters were then derived from the Arrhenius equation, which can be read as follows:

2 $$\ln k_{\mathrm{app}}=\ln A-\frac{E_{a}}{R}\times \frac{1}{T}$$

where *E*_a_ is the apparent activation energy, *R* is the universal gas constant (8.314 J/mol·K), *T* is the absolute reaction temperature, and *A* is the pre-exponential factor.^37^ Representative Arrhenius plots are shown in Figure 6, while numerical data are summarized in Table 4. At low ZnO loadings (0.25–0.50 wt %), relatively high activation energies (>90 kJ/mol) were obtained, indicating a strong temperature dependence of the polymerization rate. The corresponding Arrhenius fits exhibited excellent linearity (*R* ^2^ > 0.96), confirming the reliability of the extracted activation parameters within the investigated temperature range (Figure 6A and B). Such behavior suggested that the limited efficiency of mechanochemical reduction of Cu­(II)/L to the active Cu­(I)/L species was the main factor limiting the rate of polymerization under these conditions. In contrast, increasing the ZnO loading to 0.75–1.0 wt % resulted in a pronounced decrease of the apparent activation energy to 36.4 and 24.9 kJ/mol, respectively. Such a reduction in *E*_a_ indicated a substantially weaker temperature dependence of the polymerization rate, suggesting that the mechanochemical reduction of Cu­(II)/L to Cu­(I)/L proceeded more efficiently when a sufficient number of piezoelectrically active ZnO interfaces was present. Under these conditions, the polymerization rate was more likely governed by the dynamic activation–deactivation equilibrium characteristic of ATRP, rather than by slow Cu­(I) catalyst generation.^2,35^ Consistent with this interpretation, the *k*_app_ values exhibited reduced sensitivity to reaction temperature, particularly between 35 and 45 °C (Figure 6C and D). Consequently, the Arrhenius plots displayed only moderate linearity (*R* ^2^ ∼ 0.75–0.77), indicating a deviation from ideal Arrhenius behavior under these conditions. At the highest ZnO loading (2.0 wt %), the apparent activation energy increased again to 45.1 kJ/mol, accompanied by a decrease in polymerization efficiency compared with the optimal ZnO loading (Figure 6E). This behavior may be associated with heterogeneous effects such as nanoparticle aggregation and existence of radical concentration gradients. Although the Arrhenius analysis was based on three temperature points and the calculated activation energies therefore represent the apparent values, the observed trends clearly demonstrate that ZnO loading strongly influenced the efficiency of mechanochemical catalyst regeneration. Taken together, these results highlight 1.0 wt % of ZnO as the optimal loading within the temperature range of 25–45 °C, offering sufficient mechanochemical activation while maintaining well-controlled polymer growth under the given experimental conditions.

**Figure 5:**
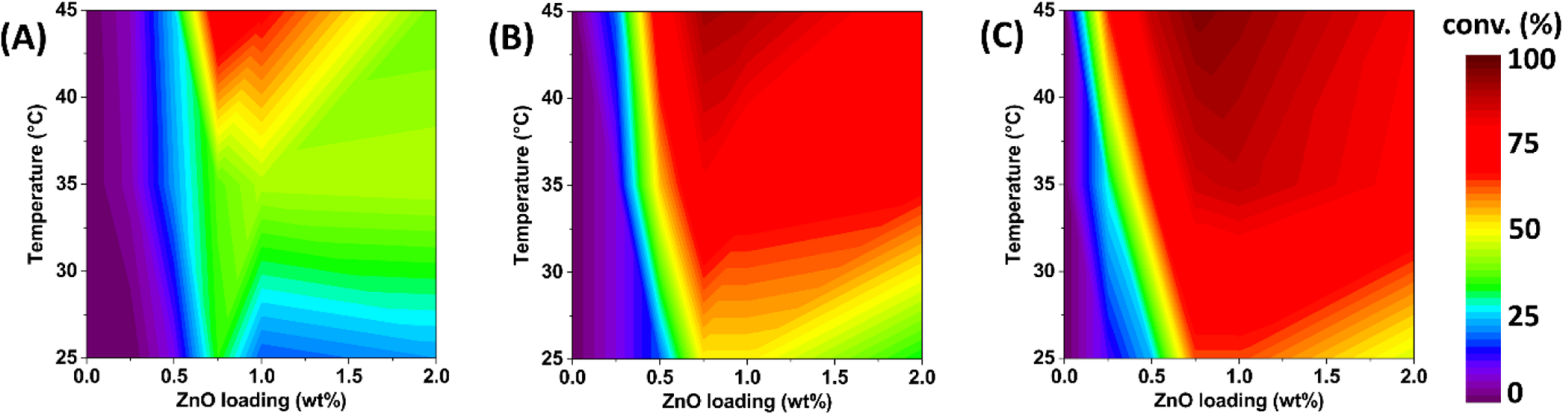
Heat maps correlating reaction temperature and ZnO loading for mechanoATRP of MA after (A) 2 h, (B) 4 h, and (C) 6 h. Reaction conditions: [MA]_0_/[EBiB]_0_/[CuBr_2_]_0_/[TPMA]_0_ = 100/1/0.04/0.16 in 50% (v/v) DMSO, argon-purged (5 min); closed-capped reactor with 20% (v/v) headspace; ultrasound source (40 kHz).

**Table 4:** Propagation Rate Constants for MechanoATRP of MA Catalyzed by Various Amounts of ZnO Nanocrystals at Different Temperatures^a^

	***k***_**app**_ **(h** ^**–1**^)			
**ZnO** (wt %)	**25 (°C)**	**35 (°C)**	**45 (°C)**	***E***_**a**_ (kJ/mol)
0.00	N.D.	N.D.	N.D.	N.D.
0.25	0.023	0.070	0.246	92.9
0.50	0.044	0.217	0.436	91.3
0.75	0.146	0.362	0.363	36.4
1.00	0.197	0.373	0.367	24.9
2.00	0.092	0.237	0.286	45.1

a Reaction conditions: [MA]_0_/[EBiB]_0_/[CuBr_2_]_0_/[TPMA]_0_ = 100/1/0.04/0.16 in 50% (v/v) DMSO, argon-purged (5 min); closed-capped reactor with 20% (v/v) headspace; ultrasound source (40 kHz).

**Figure 6:**
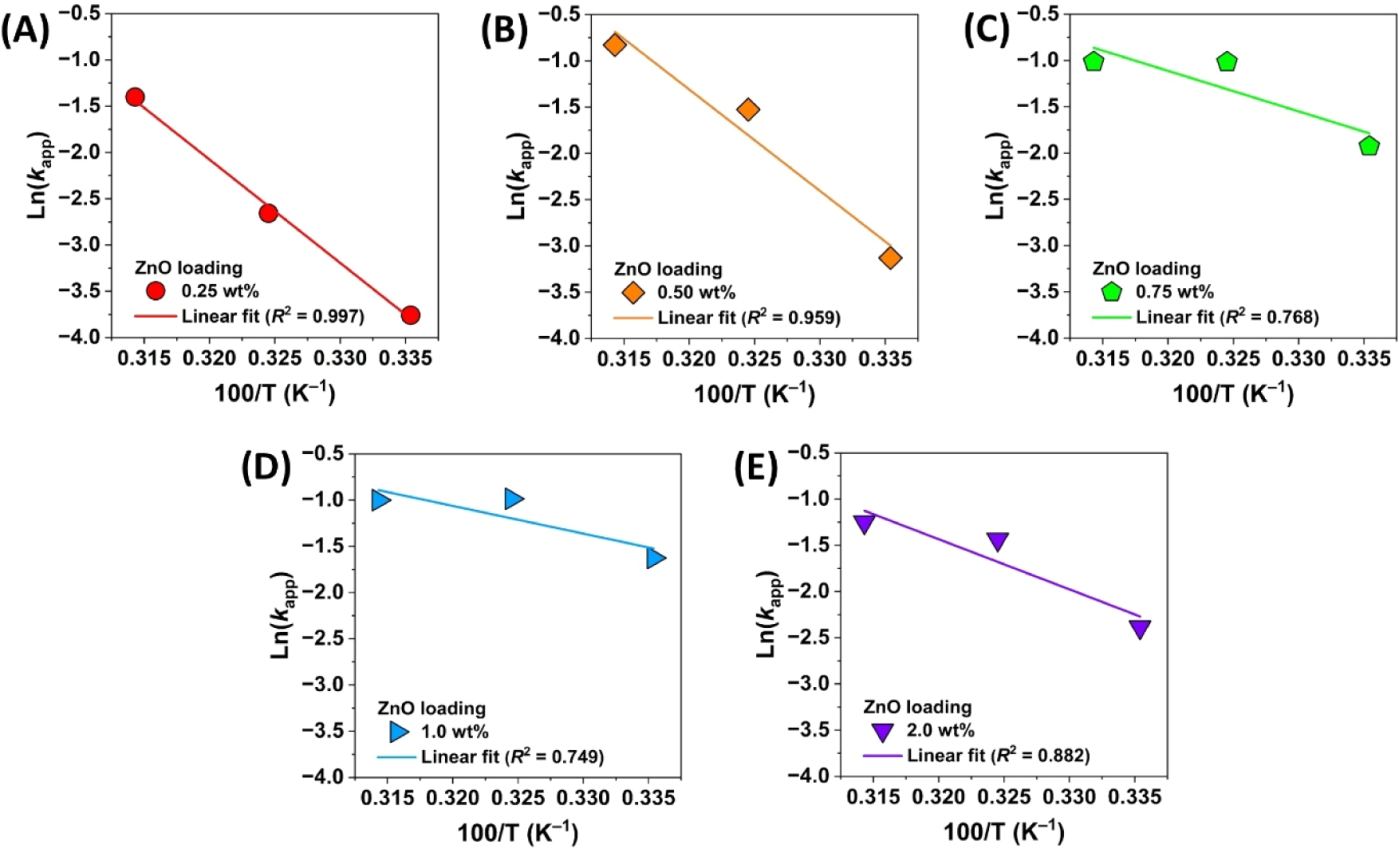
Arrhenius plots for mechanoATRP of MA performed at ZnO loadings of (A) 0.25, (B) 0.50, (C) 0.75, (D) 1.0, and (E) 2.0 wt %. The corresponding linear fits and *R* ^2^ values are indicated. For intermediate ZnO loadings (0.75 and 1.0 wt %), the polymerization rate exhibited reduced temperature sensitivity, reflected by the smaller change in *k*_app_ between 35 and 45 °C. Reaction conditions: [MA]_0_/[EBiB]_0_/[CuBr_2_]_0_/[TPMA]_0_ = 100/1/0.04/0.16 in 50% (v/v) DMSO, argon-purged (5 min); closed-capped reactor with 20% (v/v) headspace; ultrasound source (40 kHz).

## 4 Conclusions

In summary, this work systematically examined the influence of ZnO loading and reaction temperature on the kinetics and polymerization control of mechanoATRP of MA. The reactions were performed in an ultrasonic setup with precise temperature control, which enabled decoupling of thermal and mechanochemical contributions to Cu-catalyst (re)­generation, allowing quantitative assessment of the underlying mechanoredox activation process. The results demonstrated that both parameters have significant implications; low ZnO loading (≤0.5 wt %) and low temperature (25 °C) provided insufficient mechanochemical activation, leading to slow polymerization rate and extended induction period. Increasing either parameter (≥0.75 wt % of ZnO, ≥35 °C) enhanced radical generation and enabled faster yet well-controlled polymer growth. In contrast, excessive ZnO loading (2.0 wt %) impaired the mechanoATRP equilibrium, leading to a loss of control and higher *Đ*. Arrhenius analysis further revealed that the apparent activation energy strongly depended on ZnO loading, indicating a transition from a regime limited by inefficient catalyst activation at low ZnO loadings to a regime governed by the activation–deactivation equilibrium at intermediate loadings, and finally to a regime influenced by heterogeneous catalytic effects at high ZnO loadings. Kinetic analysis showed that the *k*_app_ constant was strongly temperature-dependent at low ZnO loadings, whereas higher ZnO loadings reduced sensitivity to temperature and yielded well-defined PMA (e.g., *M*_n,GPC_ of 8500 g/mol, *Đ* of 1.11, conv. of 93%, at 45 °C), corresponding to significantly lower *E*_a_ (∼25 kJ/mol). Overall, these findings established the operational boundaries of mechanoATRP of MA and identified 1.0 wt % ZnO as the optimal loading that provided a favorable balance between polymerization rate and well-controlled PMA growth. The insights gained in this study offer both mechanistic understanding and a practical basis for the rational design and scalable implementation of mechanoATRP systems.

## Supplementary Material



## Notes

Figure S1: Representative procedures for calculating monomer conversion from ^1^H NMR spectra; Figure S2: Additional GPC traces for selected ZnO loadings; Figure S3: Correlation between ZnO loading and monomer conversion at different reaction times and temperatures; Figure S4: Semilogarithmic kinetic plots used for *k*_app_ determination (PDF)
