# Supplementary material for: Optimization of MechanoATRP through ZnO Loading and Reaction Temperature
Source: ACS Polym Au. 2026 Apr 2;6(3):839–48. doi: 10.1021/acspolymersau.5c00197 (PMC13261722; doi:10.1021/acspolymersau.5c00197)
Supplement: Supplementary file 1 [file lg5c00197_si_001.pdf]

Supporting Information

Optimization of MechanoATRP through ZnO Loading and Reaction Temperature

Martin Cvek*, Dominik Skopal, Miroslav Mrlik

Centre of Polymer Systems, Tomas Bata University in Zlin, Trida T. Bati 5678, 760 01 Zlin, Czech Republic

*Author to whom correspondence should be addressed: cvek@utb.cz

Table of Contents

1. Experimental part 2

1.1. Determination of monomer conversion by ^{1}H NMR....................2

Figure S1. Representative procedures for calculating monomer conversion from ^{1}H NMR spectra. Reaction conditions: $[MA]_0/[EBiB]_0/[CuBr_2]_0/[TPMA]_0$ = 100/1/0.04/0.16 in 50% (v/v) DMSO, argon-purged (5 minutes); closed-capped reactor with 20% (v/v) headspace; ultrasound source (40 kHz) at 35°C, ZnO loading of 0.75 wt%, withdrawal time of 2 hours. 2

2. Results and Discussion....................3

2.1. Screening of mechanoATRP kinetics 3

Figure S2. The representative GPC traces for mechanoATRP of MA catalyzed with ZnO nanocrystal loadings of (A) 0.25, and (B) 2.0 wt%. Reaction conditions: $[MA]_0/[EBiB]_0/[CuBr_2]_0/[TPMA]_0$ = 100/1/0.04/0.16 in 50% (v/v) DMSO, argon-purged (5 minutes); closed-capped reactor with 20% (v/v) headspace; ultrasound source (40 kHz) at 35°C....................3

2.2. Relationship between ZnO loading and conversion 3

Figure S3. Correlation between ZnO loading and monomer conversion at different reaction times (2, 4, and 6h) and temperatures: (A) 25, (B) 35, and (C) 45°C. Reaction conditions: $[MA]_0/[EBiB]_0/[CuBr_2]_0/[TPMA]_0$ = 100/1/0.04/0.16 in 50% (v/v) DMSO, argon-purged (5 minutes); closed-capped reactor with 20% (v/v) headspace; ultrasound source (40 kHz). 3

Figure S4. Semilogarithmic kinetic plots with the fitted region (2–6 h) used for k_{app} determination highlighted, recorded at (A) 25°C and (B) 45°C. Reaction conditions: $[MA]_0/[EBiB]_0/[CuBr_2]_0/[TPMA]_0$ = 100/1/0.04/0.16 in 50% (v/v) DMSO, argon-purged (5 minutes); closed-capped reactor with 20% (v/v) headspace; ultrasound source (40 kHz). 4

Supporting Information

1. Experimental part

1.1. Determination of monomer conversion by ^{1}H NMR

Monomer conversion was determined by ^{1}H NMR spectroscopy by comparing the integral of the vinyl proton signal of the monomer (–CH=CH_2) at approximately 5.7 ppm with the broad signal corresponding to the methine proton of the polymer backbone (–CH–) at 2.3–2.2 ppm in poly(methyl acrylate) (PMA) (**Figure S1A**). These results were further verified by comparing the signals corresponding to the methoxy protons of the ester group (–OCH_3) in the monomer and polymer at approximately 3.8 ppm and 3.6 ppm, respectively (**Figure S1B**).

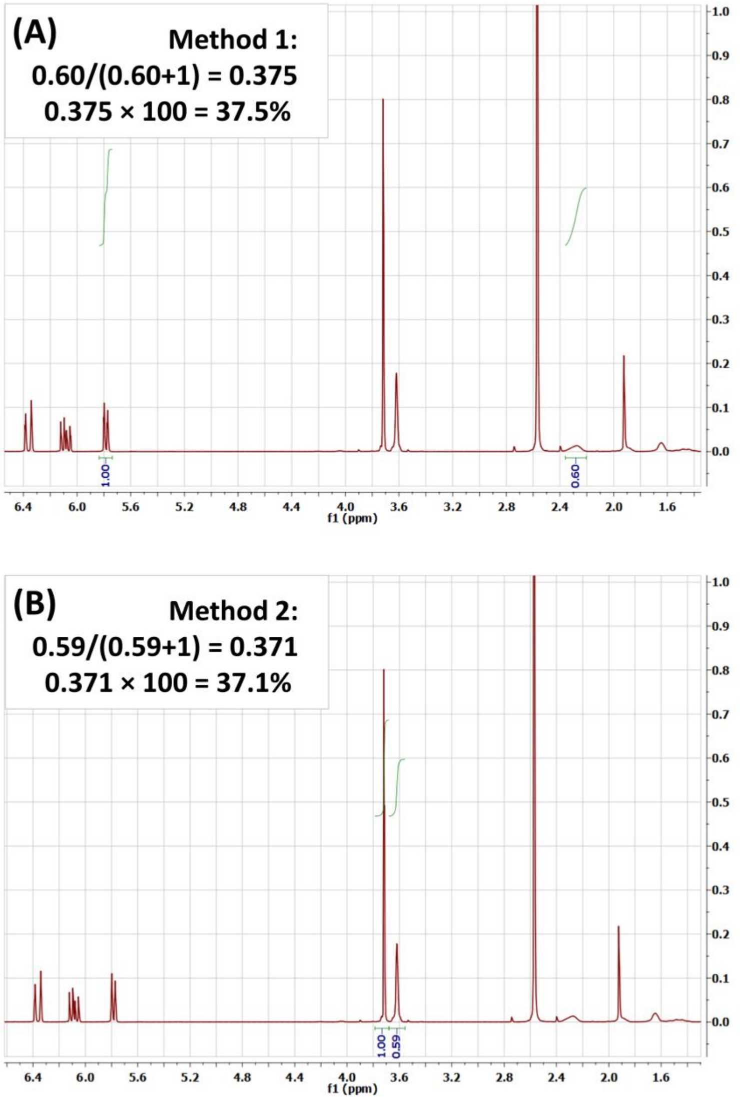

Figure S1. Representative procedures for calculating monomer conversion from ^{1}H NMR spectra. Reaction conditions: $[MA]_0/[EBiB]_0/[CuBr_2]_0/[TPMA]_0$ = 100/1/0.04/0.16 in 50% (v/v) DMSO, argon-purged (5 minutes); closed-capped reactor with 20% (v/v) headspace; ultrasound source (40 kHz) at 35°C, ZnO loading of 0.75 wt%, withdrawal time of 2 hours.

Supporting Information

2. Results and Discussion

2.1. Screening of mechanoATRP kinetics

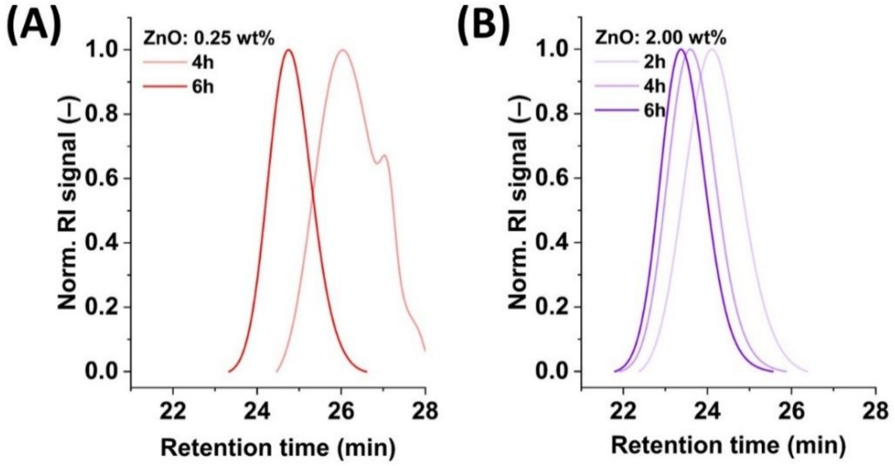

Figure S2. The representative GPC traces for mechanoATRP of MA catalyzed with ZnO nanocrystal loadings of (A) 0.25, and (B) 2.0 wt%. Reaction conditions: $[MA]_0/[EBiB]_0/[CuBr_2]_0/[TPMA]_0$ = 100/1/0.04/0.16 in 50% (v/v) DMSO, argon-purged (5 minutes); closed-capped reactor with 20% (v/v) headspace; ultrasound source (40 kHz) at 35°C.

2.2. Relationship between ZnO loading and conversion

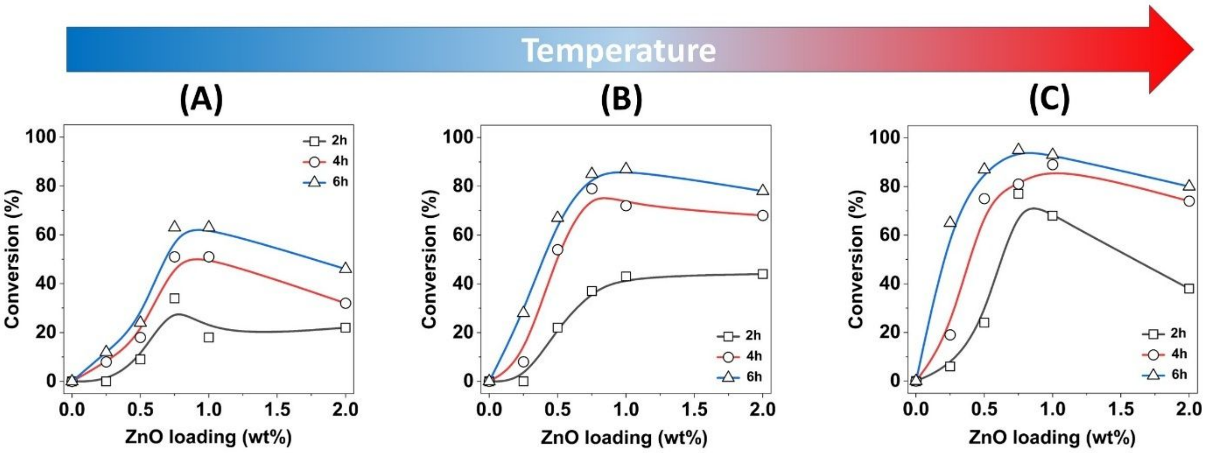

Figure S3. Correlation between ZnO loading and monomer conversion at different reaction times (2, 4, and 6h) and temperatures: (A) 25, (B) 35, and (C) 45°C. Reaction conditions: $[MA]_0/[EBiB]_0/[CuBr_2]_0/[TPMA]_0$ = 100/1/0.04/0.16 in 50% (v/v) DMSO, argon-purged (5 minutes); closed-capped reactor with 20% (v/v) headspace; ultrasound source (40 kHz).

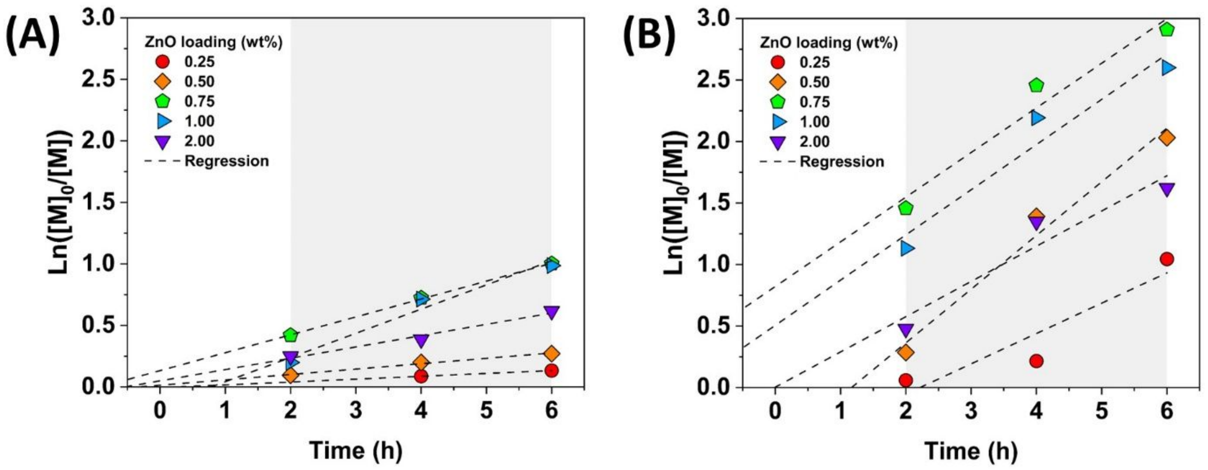

Figure S4. Semilogarithmic kinetic plots with the fitted region (2–6 h) used for k_{app} determination highlighted, recorded at (A) 25 and (B) 45°C. Reaction conditions: $[MA]_0/[EBiB]_0/[CuBr_2]_0/[TPMA]_0$ = 100/1/0.04/0.16 in 50% (v/v) DMSO, argon-purged (5 minutes); closed-capped reactor with 20% (v/v) headspace; ultrasound source (40 kHz).
